# Cardiac events after using clarithromycin for anti-*Helicobacter pylori* therapy in patients with coronary artery disease

**DOI:** 10.1097/MD.0000000000035922

**Published:** 2023-11-10

**Authors:** Cheng-Kuan Lin, Tzong-Hsi Lee, Pen-Chih Liao, Cheng-Lu Lin

**Affiliations:** a Division of Gastroenterology and Hepatology, Department of Internal Medicine, Far Eastern Memorial Hospital, Banqiao District, New Taipei City, Taiwan; b Division of Cardiology, Center of Cardiovascular Medicine, Far Eastern Memorial Hospital, Banqiao District, New Taipei City, Taiwan.

**Keywords:** clarithromycin, coronary artery disease, diabetes, *Helicobacter pylori*, peptic ulcer

## Abstract

Clarithromycin is an antibiotic commonly used to treat *Helicobacter pylori* infections. The US Food and Drug Administration (FDA) advises caution before prescribing clarithromycin to patients with cardiac diseases. This study aimed to evaluate cardiac events after anti-*H pylori* treatment in patients with coronary artery disease. A retrospective 5-year study was conducted on outpatients who received anti-*H pylori* therapy. Among the 7855 patients receiving therapy, 228 patients (2.9%) underwent angiography with coronary artery disease before therapy, and 193 patients received clarithromycin. Clarithromycin users seemed not to be at risk for cardiac events as compared with non-clarithromycin users at 3 months (4.7% vs 2.9%, *P* = .63) and 1 year (10.9% vs 5.7%, *P* = .35). Neither life-threatening dysrhythmia nor cardiac death was noted. The risk factors for cardiac events within 3 months after therapy were smoker (OR:5.38, 95% CI:1.39–20.78), and events within 1 year were smoker (OR:3.8, 95% CI:1.41–10.22), and diabetes mellitus (OR:5.68, 95% CI:1.9–16.98). Among patients with coronary artery disease who received anti-*H pylori* therapy, short-term cardiac events did not increase in clarithromycin users but should be considered in diabetic and smoking patients.

## 1. Introduction

*Helicobacter pylori* infection is the most common cause of peptic ulcer disease, and its eradication reduces the recurrence of peptic ulcers. Clarithromycin is an important antibiotic used in anti-*H pylori* regimens. On February 22, 2018, the US Food and Drug Administration (FDA) advised caution in prescribing clarithromycin to patients with cardiac disease, based on a study that showed an increased short-term risk of cardiac events or death.^[1,2]^ Three years of follow-up data from the large CLARICOR randomized placebo-controlled trial revealed short-term clarithromycin use in patients with stable coronary heart disease associated with increased cardiovascular mortality. Patients with ischemic heart diseases are predisposed to have peptic ulcers, and anti-*H pylori* therapy is indicated for the prevention of recurrence when the bacteria are detected. Therefore, the safety of clarithromycin for anti-*H pylori* infection in patients with coronary artery disease is an important issue before prescribing medications. We conducted a hospital-based retrospective study to investigate cardiac events after anti-*H pylori* therapy in patients with angiography-proven coronary artery diseases. Risk factors for cardiac events after therapy were also analyzed.

## 2. Material and methods

### 2.1. Study design

A hospital-based retrospective chart review was conducted for patients aged ≥ 20 years who underwent anti-*H pylori* therapy in an outpatient clinic at Far Eastern Memorial Hospital, Taiwan from January 2013 to December 2017. Patients diagnosed with coronary artery disease by finding stenotic vessels on coronary angiography were enrolled in this study. The exclusion criteria were incomplete therapy due to allergy, and a history of macrolide use before 3 months of therapy.

### 2.2. Data collection

Patient demographic data, including age, sex, and smoking status were collected. Patients with underlying diseases, such as hypertension, diabetes mellitus, hyperlipidemia, chronic renal failure, and stroke were recorded. Hyperlipidemia was defined as a serum total cholesterol level > 200 mg/dL or triglyceride level > 200 mg/dL. Chronic renal failure was defined as an estimated glomerular filtration rate <20%. The severity of coronary artery disease was graded by the number of vessels with arterial stenosis in the coronary angiogram. Significant coronary artery disease was defined as >50% stenosis of the left main artery, or >70% stenosis of either 3 coronary vessels. Interventions for significant coronary artery disease included balloon angioplasty, stenting, and bypass grafting surgery. Associated heart diseases, such as cardiac arrhythmia detected on electrocardiogram, severe valvular disease noted on echocardiography, and heart failure with left ventricular ejection fraction <40% were recorded. Arrhythmia was documented in previous chart history, and by electrocardiogram when suspicious clinical symptoms/signs, or during admission routine checkups. Medications including antiplatelet agents, anticoagulants, antiarrhythmic drugs, and statins were recorded. We reviewed concomitant drugs that may lead to QT prolongation, such as macrolides, quinolones, antiarrhythmics, antidepressants, and antipsychotics. The regimen of antimicrobial agents, proton pump inhibitors, and duration of anti-*H pylori* therapy were recorded.

### 2.3. Outcome measurement

Patient outcomes included cardiac events and cardiovascular and all-cause mortality after clarithromycin-based or non-clarithromycin-based therapy. Cardiac events were defined as unstable angina, acute myocardial infarction, or dysrhythmia requiring hospital admission for intervention within 3 months and 1 year after therapy. Risk factors for developing cardiac events after anti-*H pylori* therapy were analyzed.

### 2.4. Statistical analysis

The study results were expressed as median, standard deviation, range, case numbers, and percentages. Numerical data were compared using the Mann-Whitney U-test, and categorical data were compared using the chi-square test. Logistic regression analysis was used to predict risk factors for cardiac events after therapy. A univariate analysis was performed for each independent variable. The variables were selected for multivariate analysis if they were significant with a *P* value < .20. A *P* value of <.05 was considered the statistical significance and all tests were 2-tailed. All statistical analyses were performed using the Stata software (version 13.0; Stata Corp., College Station, TX).

### 2.5. Statement of ethics

This study was conducted in accordance with the ethical standards of the Declaration of Helsinki. The study protocol was approved by the Research Ethics Review Committee of the Far Eastern Memorial Hospital.

## 3. Results

### 3.1. Patient characteristics

In total, 7884 patients received anti-*H pylori* therapy within the 5-year study period. Twenty-nine patients were excluded because of incomplete treatment (21 patients) or previous macrolide use (8 patients). Among the 7855 patients with complete treatment, 228 (2.9%) with stenotic coronary angiograms were enrolled. After first-line anti-*H pylori* therapy, 25 patients received second-line therapy and 3 received third-line therapy with triple therapy, levofloxacin-based therapy, dual therapy, or sequential therapy. One hundred and ninety-three patients received clarithromycin-based triple therapy.

The median age of the patients was 65 ± 10.3 years old (range, 32–90 years), and male patients were predominant (73.7%). Regarding underlying diseases, 96.1% of patients had hypertension, 47.4% had diabetes mellitus, 54.8% had hyperlipidemia, and 9.6% had chronic renal failure. Twenty-one percent of the patients were smokers and 33.3% of the patients had ulcer bleeding. The grades of coronary artery disease were 1-vessel disease (24.1%), 2-vessel disease (26.3%), 3-vessel disease (48.2%), and the left main disease (1.3%). The coronary artery interventions included balloon angioplasty (6.6%), artery stenting (63.2%), and coronary artery bypass graft surgery (18.4%). Three percent of the patients had arrhythmia, 2.2% had severe valvular heart disease, and 4.8% had congestive heart failure. Antiplatelet agents or anticoagulants were used in 86% of the patients. Sixty percent of the patients received statins as lipid-lowering agents and 3.1% of the patients received antiarrhythmic drugs, but none of these drugs led to QT prolongation. The median duration of anti-*H pylori* therapy was 7 ± 1.7 days (range: 7–14 days).

### 3.2. Patient outcomes

The patients’ characteristics, medications, and status of the clarithromycin or non-clarithromycin groups were listed. (Table 1) The non-clarithromycin group used more metronidazole, tetracycline, and bismuth than the clarithromycin group did. Increased statin use in the non-clarithromycin group was attributed to the doctor concern about the possible interaction between clarithromycin and statin.

**Table 1:** The characteristics, medications, status, and outcome in coronary artery disease patients using anti-*Helicobacter pylori* therapy.

	Clarithromycin-based regimens, N = 193 (S.D. or percentage)	Non-clarithromycin-based regimens, N = 35 (S.D. or percentage)	*P* value
Age (yr)	65 (7.5)	61 (8.8)	.24
Sex (male)	142 (73.6%)	26 (74.3%)	.93
Smoker	42 (21.8%)	6 (17.1%)	.54
Hypertension	185 (95.9%)	34 (97.1%)	.72
3-V-D and LM-D	98 (50.8%)	15 (42.9%)	.39
Arrhythmia	6 (3.1%)	0	.29
VHD	3 (1.6%)	2 (5.7%)	.12
Heart failure	9 (4.7%)	2 (5.7%)	.79
DM	91 (47.2%)	17 (48.6%)	.88
Hyperlipidemia	113 (58.5%)	22 (62.9%)	.63
Renal failure	19 (9.8%)	3 (8.6%)	.81
Stroke	9 (4.7%)	1 (2.9%)	.63
Use of esomeprazole	77 (39.9%)	12 (34.3%)	.69
Clopidogrel	95 (49.2%)	16 (45.7%)	.70
Statin	111 (57.5%)	27 (77.1%)	.03*
Amoxicillin	192 (99.5)	34 (97.1)	.17
Metronidazole	7 (3.6)	13 (37.1)	<.01*
Tetracycline	1 (0.5)	3 (8.6)	<.01*
Bismuth	0	12 (34.3)	<.01*
Anti-*H pylori* duration (d)	7 (1.6)	8 (2.4)	.07
Anti-*H pylori* > 1 time	22 (11.4%)	3 (8.6%)	.63
Ulcer bleeding	67 (34.7%)	9 (25.7%)	.17
s/p CABG	33 (17.1%)	9 (25.7%)	.23
s/p Stenting	123 (63.7%)	21 (60%)	.67
Intervention before 3 mo	8 (4.1%)	3 (8.6%)	.26

3-V-D = 3-vessel disease, CABG = coronary artery bypass grafting, CAD = coronary artery disease, LM-D = left main disease, S.D. = standard deviation, VHD = valvular heart disease.

Ten patients developed cardiac events within 3 months after therapy. In the clarithromycin group, 8 patients had unstable angina and 1 patient had an acute myocardial infarction. One patient in the non-clarithromycin group developed unstable angina. Twenty-three patients developed cardiac events within 1 year after treatment. In the clarithromycin group, 18 patients had unstable angina and 3 patients had acute myocardial infarctions. Two patients who did not receive clarithromycin developed unstable angina. These events did not occur during the antibiotic use.

There were no significant increases in cardiac events in clarithromycin users compared to non-clarithromycin users (within 3 months: 4.7% vs 2.9%, *P* = .63; within 1 year: 10.9% vs 5.7%, *P* = .35). For the clarithromycin users with the duration of 2 weeks therapy (23.8% of the patients) compared with less than 2 weeks therapy, the cardiac events were not significantly increased (within 3 months: 0% vs 6.1%, *P* = .09; within 1 year: 4.3% vs 12.9%, *P* = .10).

No life-threatening dysrhythmia or cardiac death was noted within 1 year of anti-*H pylori* therapy. Four patients had non-cardiac mortality within 1 year after treatment. One patient in the clarithromycin group had an intracranial hemorrhage, and 1 patient had sepsis. One patient in the non-clarithromycin group had colon cancer and 1 patient had pneumonia.

Logistic regression analysis of risk factors for cardiac events within 3 months and 1 year after anti-*H pylori* therapy revealed that clarithromycin user was not associated with this risk. The factors with the highest odds for cardiac events within 3 months after therapy were smokers (odds ratios [OR]:5.38, 95% confidence intervals [CI]:1.39–20.78; *P* = .02), and the highest odds for cardiac events within 1 year were smokers (OR: 3.8, 95% CI:1.41–10.22; *P* = .01), and diabetes mellitus (OR: 5.68, 95% CI: 1.9–16.98; *P* < .01) (Table 2). Age, sex, hyperlipidemia, previous myocardial infarction, arrhythmia, renal failure, and type of proton pump inhibitors were not risk factors.

**Table 2:** Risk factors for cardiac events after anti-*Helicobacter pylori* therapy.

	Univariate analysis			Multivariate analysis		
	Odds ratio	95% CI	*P* value	Odds ratio	95% CI	*P* value
Cardiac events within 3 mo						
Clarithromycin Levofloxacin Age (≥70) Sex (male) Smoker	1.66 3.16 1.1 1.45 4.07	0.2–13.56 0.62–16.12 0.3–4.03 0.3–7.02 1.13–14.69	.64 .17 .88 .65 .03*	5.38	1.39–20.78	.02*
DM	4.72	0.98–22.74	.05	4.52	0.88–23.09	.07
Hyperlipidemia	1.64	0.41–6.52	.48			
Renal failure	2.48	0.49–12.46	.27			
Previous MI	0.23	0.28–1.81	.26			
3-V-D/LM-D	1.56	0.43–5.67	.50			
Arrhythmia	4.73	0.5–44.83	.28			
Esomeprazole	4.19	1.05–16.66	.04*	3.92	0.92–16.63	.06
Statin	0.98	0.27–3.56	.97			
Cardiac events within 1 yr						
Clarithromycin	2.01	0.45–9.01	.36			
Levofloxacin	1.12	0.24–5.23	.88			
Age (≥70)	0.7	0.27–1.77	.45			
Sex (male)	0.64	0.26–1.59	.33			
Smoker	2.74	1.1–6.78	.03*	3.8	1.41–10.22	.01*
DM	4.6	1.04–12.87	<.01*	5.68	1.9–16.98	<.01*
Hyperlipidemia	1.65	0.65–4.19	.29			
Renal failure	2.19	0.67–7.13	.19	1.17	0.33–4.18	.81
Previous MI	0.73	0.27–1.43	.52			
3-V-D/LM-D	1.37	0.57–3.25	.48			
Arrhythmia	1.82	0.2–16.27	.59			
Esomeprazole	1.33	0.56–3.19	.52			
Statin	0.83	0.35–1.99	.68			

3-V-D = 3-vessel disease, LM-D = left main disease, MI = myocardial infarction.

After the median follow-up of 58 months (range, 0.3–103.4 months), 46 patients (23.8%) developed cardiac events at the median of 20 months after therapy. Six patients (3.1%) died of cardiac diseases, and 19 (9.8%) died of non-cardiac diseases. The cumulative incidence of cardiac events during follow-up was greater in diabetic patients than in nondiabetic patients (log-rank test *P* = .04). No significant increases were noted in smoking patients compared to nonsmoking patients (log-rank test *P* = .81).

## 4. Discussion

This study enrolled patients with significant coronary artery disease who had received anti-*H pylori* therapy before the US FDA statement warning. The results showed a trend of increasing cardiac events after clarithromycin therapy. There was no significant difference between clarithromycin and non-clarithromycin users. There might be a weak risk between exposure to clarithromycin and subsequent short-term cardiac events. The use of clarithromycin, which led to dysrhythmia was not observed. Cardiac events within 3 months increased in the patients who smoked, and those diabetic patients also had increased cardiac events within 1 year after treatment and longer follow-up. The FDA advises caution before prescribing clarithromycin for patients with heart disease. Based on our results, the risks should be monitored in patients at risk of coronary atherosclerosis.

Smoking and diabetes mellitus are well-known risk factors for coronary atherosclerosis. Those are established risks for the development and progression of coronary atherosclerosis and are associated with increased cardiac events after revascularization. Diabetes mellitus and smoking were predictors of coronary restenosis after stent implantation and the risk of reintervention after coronary bypass grafting.^[3,4]^

Although esomeprazole use was a risk factor for cardiac events within 3 months after anti-*H pylori* therapy by univariate analysis, but not a risk factor by multivariate analysis. Interaction of esomeprazole with hepatic cytochrome *P*-450 system had proposed to decrease the effect of clopidogrel, then increase the cardiac events. We found that combined esomeprazole and clopidogrel use did not increase the cardiac events within 3 months after therapy (combined clopidogrel use: 6.7% vs no clopidogrel use: 10.7%, *P* = .54).

The use of clarithromycin for anti-*H pylori* therapy has been reported to increase the incidence of cardiac events. In a Hong Kong population study that excluded patients with preexisting myocardial infarction, arrhythmia, and stroke, the incidence of myocardial infarction within 14 days after clarithromycin use was 1.28 per 1000 persons, and arrhythmia was 0.21 per 1000 persons. The risk of clarithromycin use compared with amoxicillin use with increased incidence rate ratios for myocardial infarction (3.66, 95% CI:2.82–4.76) and arrhythmia (2.22, 95% CI:1.22–4.06). After analysis, there was also an association between the current use of *H pylori* eradication treatment containing clarithromycin and cardiovascular events.^[5,6]^ However, this was not a real situation with the combined use of clarithromycin and amoxicillin for anti-*H pylori* therapy. Another British study excluding those who had myocardial infarction before 1 year showed no increase in the incidence rate ratio of the first myocardial infarction within 90 days after clarithromycin use.^[7]^

In the CLARICOR trial, 2 weeks of use of clarithromycin for Chlamydia pneumoniae infection in patients with stable coronary heart disease may cause higher cardiovascular mortality (hazard ratio: 1.27, 95% CI:1.03–1.54) and all-cause mortality (1.45, 95% CI:1.09–1.92) for up to 3 years.^[2]^ During 10 years of follow-up, all-cause mortality increased and was restricted to patients not receiving statins at entry, especially during the first 3 years.^[6]^ The clarithromycin use in the acute exacerbations of chronic obstructive pulmonary disease and community-acquired pneumonia increased acute coronary syndrome (1.67, 95% CI:1.04–2.68) within 1 year. Longer use for ≥ 7 days was associated with more cardiovascular events.^[8]^ Another study used clarithromycin in patients with acute myocardial infarction, and the odds ratio indicated an increased risk (1.68, 95% CI:1.38–2.03) for cardiac events or death.^[9]^

The mechanism of the association between clarithromycin and cardiovascular events is proposed. The antibiotic may activate macrophage by releasing bacterial proteins, leading to the destabilization of coronary artery plaques and acute coronary syndrome, and statin drugs may counteract these effects.^[10,11]^ Another important issue is that the administration of macrolides is associated with an increased risk for QT prolongation, tachyarrhythmia, and cardiovascular death.^[12]^

Previous studies have reported conflicting results regarding the association between the use of antibiotic use and increased mortality after anti-*H pylori* therapy. A British retrospective cohort study of patients with no myocardial infarction or stroke for 90 days before receiving antibiotics for *H pylori* eradication, reported higher long-term hazard ratios for mortality (1.09, 95% CI: 1–1.08) following outpatient use of clarithromycin versus metronidazole regimens.^[13]^ Danish patients with ischemic heart disease treated with clarithromycin for *H pylori* eradication showed no increased risk for all-cause mortality within 5 years of follow-up compared to patients not undergoing therapy.^[14]^ Another Taiwanese database study found no relationship between the dose and duration of clarithromycin-based therapy for *H pylori* eradication and mortality, and no increased risk of arrhythmia during the follow-up period.^[15]^ A meta-analysis suggested no significant association between clarithromycin use and long-term all-cause mortality, and there were no additional risks of cardiac mortality, acute myocardial infarction, and arrhythmia.^[16]^

In this study, we enrolled patients with preexisting significant coronary artery disease to clarify the risk of cardiac events after therapy. After a median of 7 days of clarithromycin use for anti-*H pylori* therapy, cardiac risk, and mortality did not significantly increase in non-clarithromycin users.

In an international study of outpatients with atherothrombosis within 1 year, 6.44% of patients with established coronary artery disease developed unstable angina leading to hospitalization and 1.44% of patients developed nonfatal myocardial infarction.^[17]^ In the CLARICOR trial, 7.4% of the patients with stable coronary artery disease developed myocardial infarction/unstable angina after clarithromycin therapy.^[2]^ In the present study, 9.3% of patients developed unstable angina, and 1.6% of patients developed myocardial infarction within 1 year after using clarithromycin for anti-*H pylori* therapy. There were 29 patients (0.4%) developing cardiac events after *H pylori* eradication treatment in 7627 patients without coronary artery disease. The incidence rate of unstable angina after clarithromycin therapy seemed to increase compared to the historical data. Further comparative studies are needed to evaluate the outcomes of the cohort with coronary artery disease who had received anti-*H pylori* therapy and those who did not.

The limitations of this study are its retrospective design and the result from only a single hospital database with small case numbers, which would limit inferences of causality and the extent to which results can be generalized to other institutions or populations. The enrolled patients were limited to those with stenotic coronary angiograms. Some patients with mild coronary artery disease may be underdiagnosed if invasive angiography is not performed. The influence of changes in smoking behavior and glycemic control in diabetes mellitus on cardiac events also needs to be clarified during follow-up and in an additional study. Further prospective randomized, controlled trials are warranted to compare the safety of clarithromycin and non-clarithromycin regimens for anti-*H pylori* treatment in high-risk patients with coronary artery disease.

## 5. Conclusions

Cardiac events did not increase after short-term use of clarithromycin for anti-*H pylori* therapy in patients with coronary artery disease. If a patient indicates receiving anti-*H pylori* therapy with clarithromycin, close monitoring after therapy is mandatory in patients with a risk of atherosclerosis who smoked and those with diabetes mellitus.

## Author contributions

**Conceptualization:** Cheng-Kuan Lin, Tzong-Hsi Lee, Pen-Chih Liao, Cheng-Lu Lin.

**Data curation:** Cheng-Kuan Lin, Tzong-Hsi Lee, Cheng-Lu Lin.

**Formal analysis:** Cheng-Kuan Lin.

**Methodology:** Cheng-Kuan Lin, Cheng-Lu Lin.

**Writing – original draft:** Cheng-Kuan Lin.

**Writing – review & editing:** Cheng-Kuan Lin, Tzong-Hsi Lee, Pen-Chih Liao, Cheng-Lu Lin.
